# Supplementary material for: Protein-Based Mechanism of Wheat Growth Under Salt Stress in Seeds Irradiated with Millimeter Waves
Source: Int J Mol Sci. 2024 Dec 30;26(1):253. doi: 10.3390/ijms26010253 (PMC11720253; doi:10.3390/ijms26010253)
Supplement: Supplementary file 1 [file ijms-26-00253-s001.zip › Supplemental Figures.pdf]

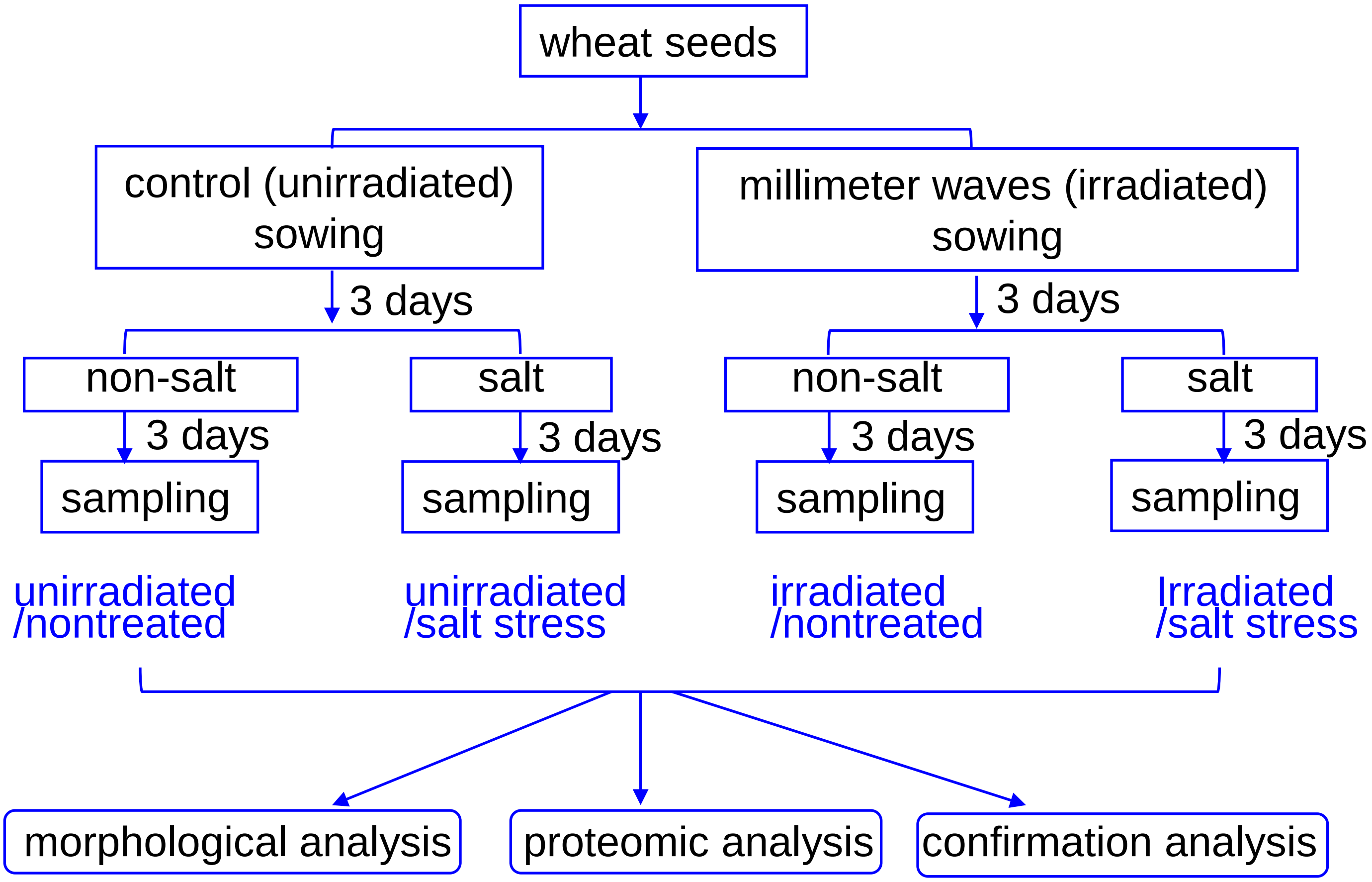

Figure S1: Experimental design for the study of the effect of MMW irradiation on wheat growth under salt stress.
Wheat seedlings were investigated with morphological, proteomic, and confirmation analyses. All experiments were performed with 3 independent biological replicates.

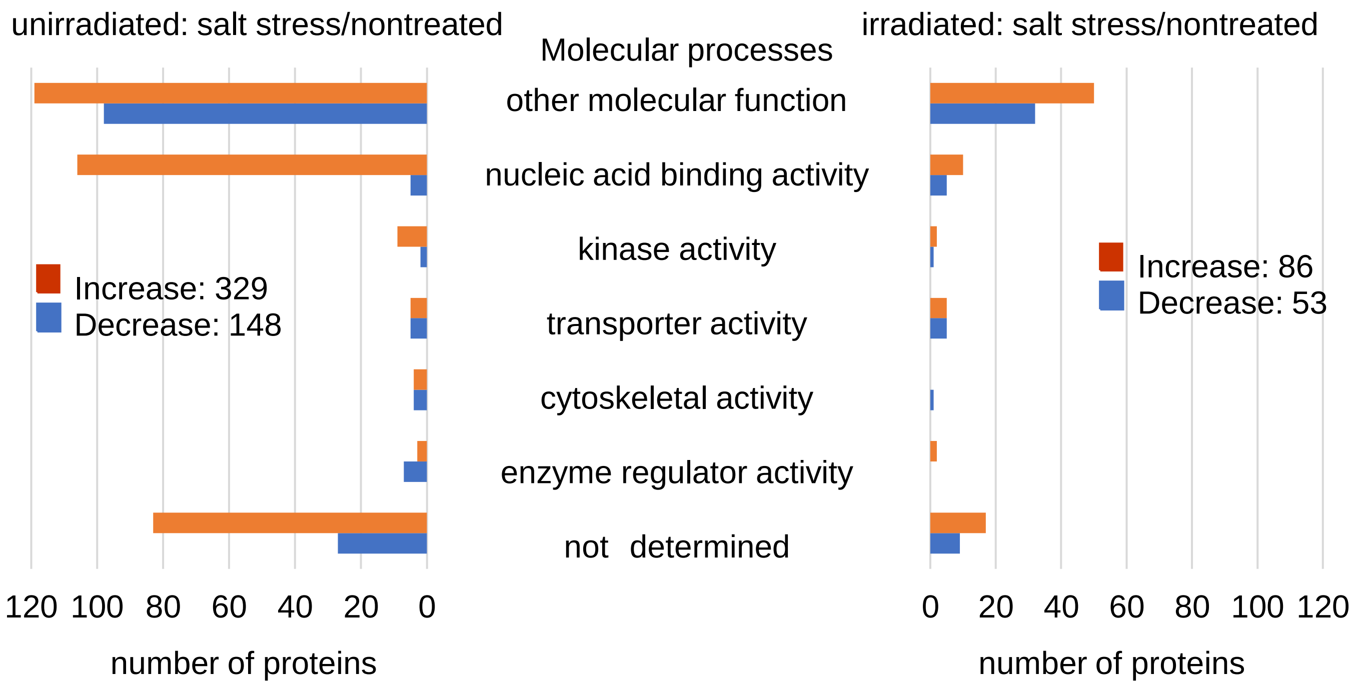

Figure S2: Molecular processes of changed proteins between unirradiated and irradiated wheat under salt stress compared with control.

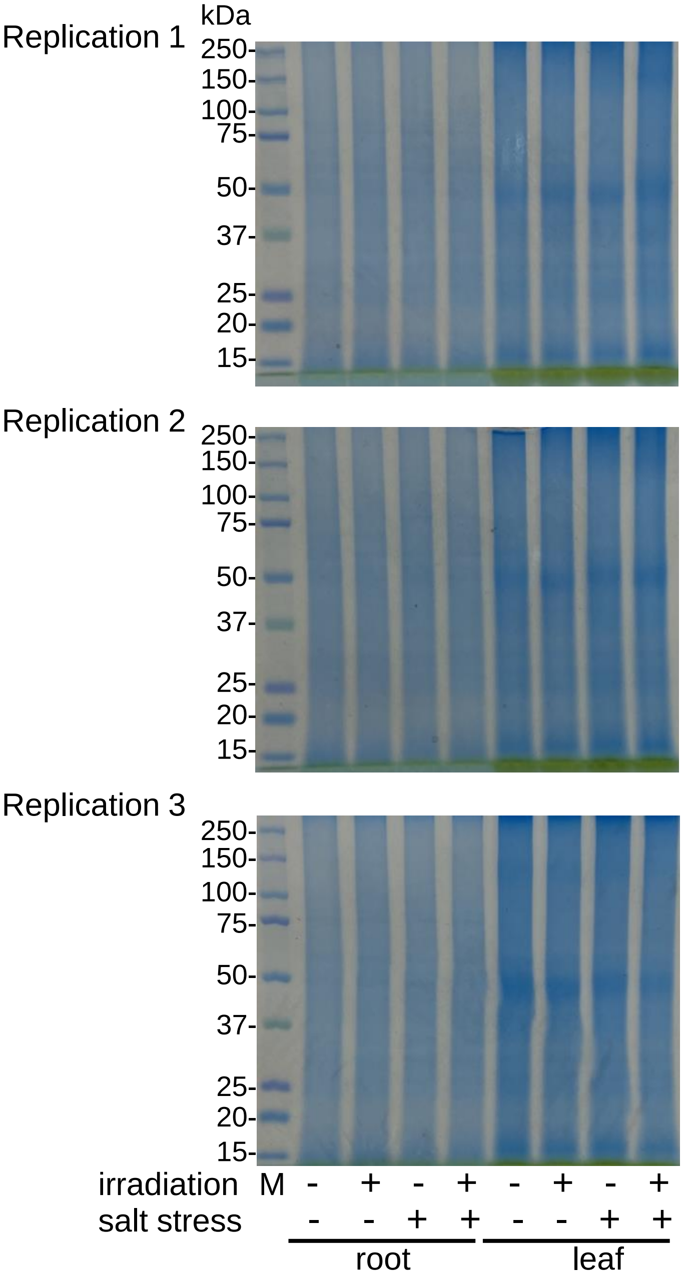

Figure S3: The Coomassie brilliant blue staining pattern of proteins used for immunoblot analysis. Experiments were performed with biologically triplicates for each treatments. Quantified proteins (10 µg) from leaf and root were separated by electrophoresis on a 10% SDS-polyacrylamide. Coomassie brilliant blue staining was used as loading control. “M” means marker proteins.

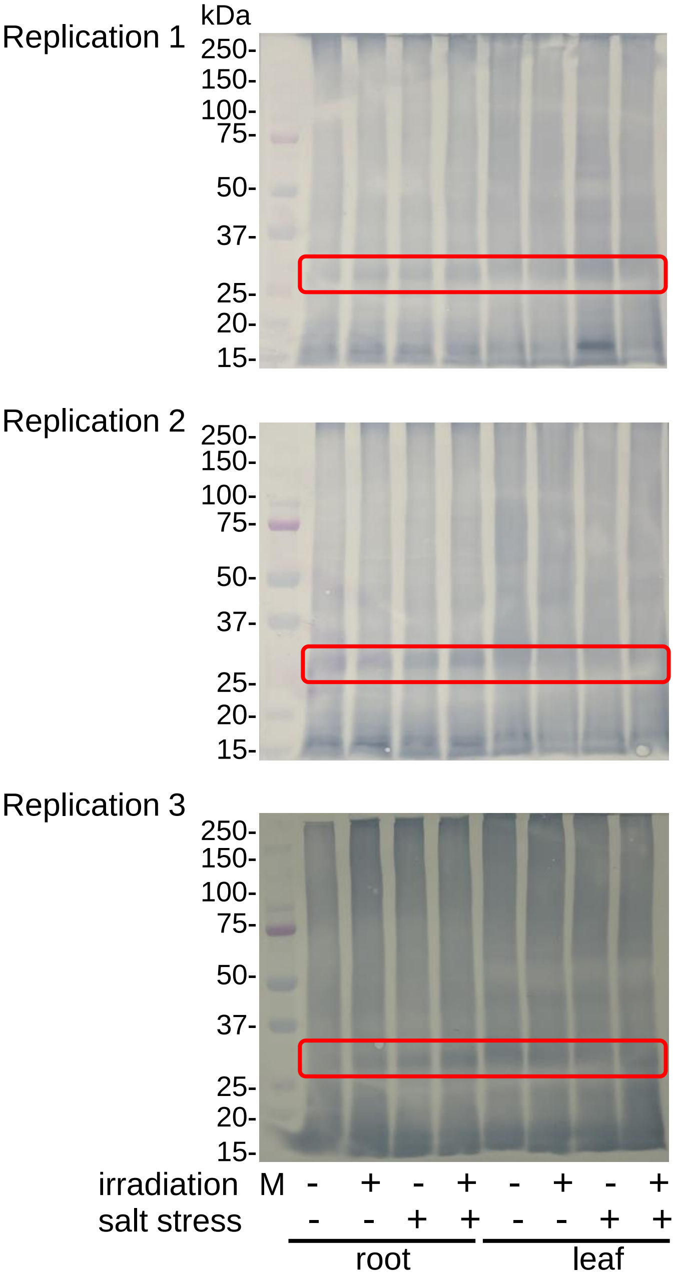

Figure S4. Blots of the entire membrane with anti-SOD antibody, which is used in Figure 4.

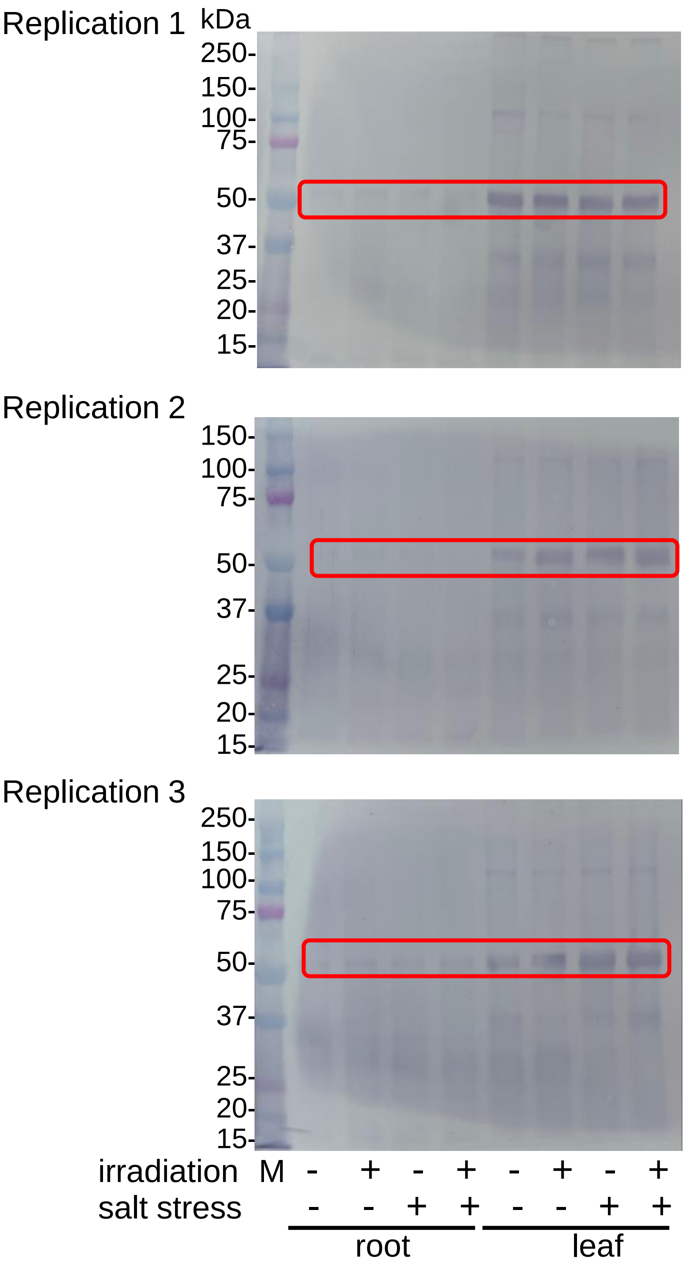

Figure S5. Blots of the entire membrane with anti-GR antibody, which is used in Figure 4.

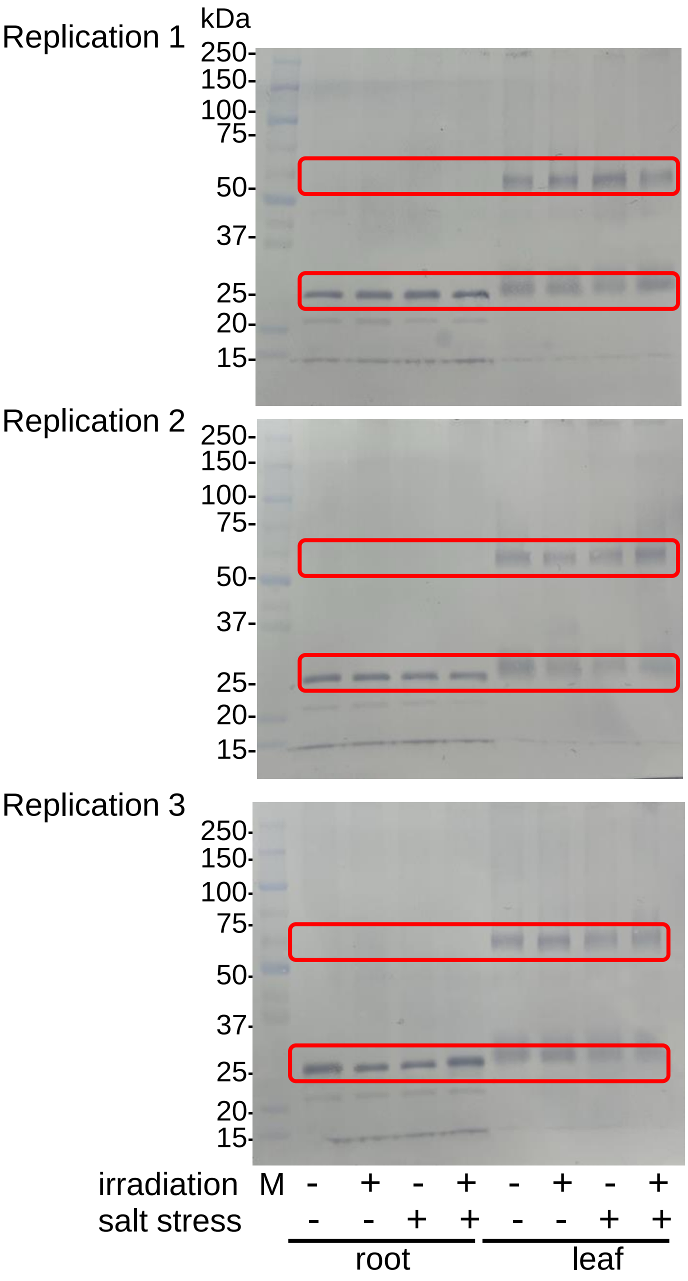

Figure S6. Blots of the entire membrane with anti-APX antibody, which is used in Figure 4.

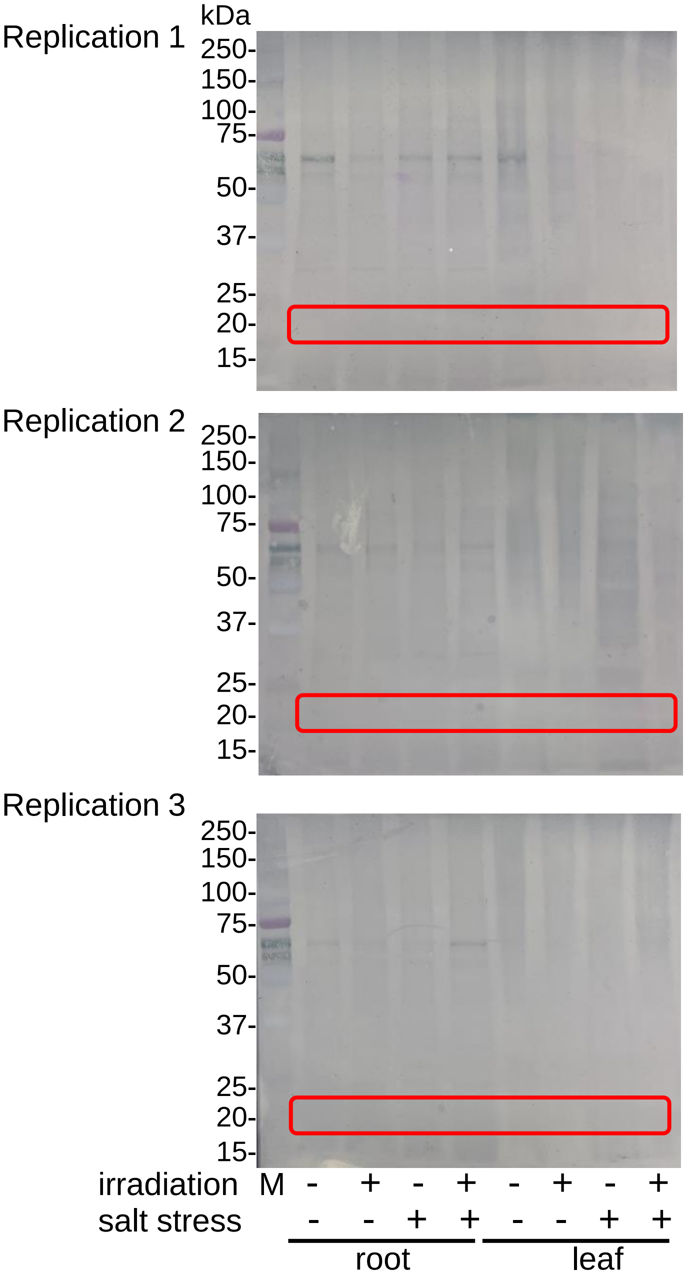

Figure S7. Blots of the entire membrane with anti-pathogenesis related protein 1 antibody, which is used in Figure 5.

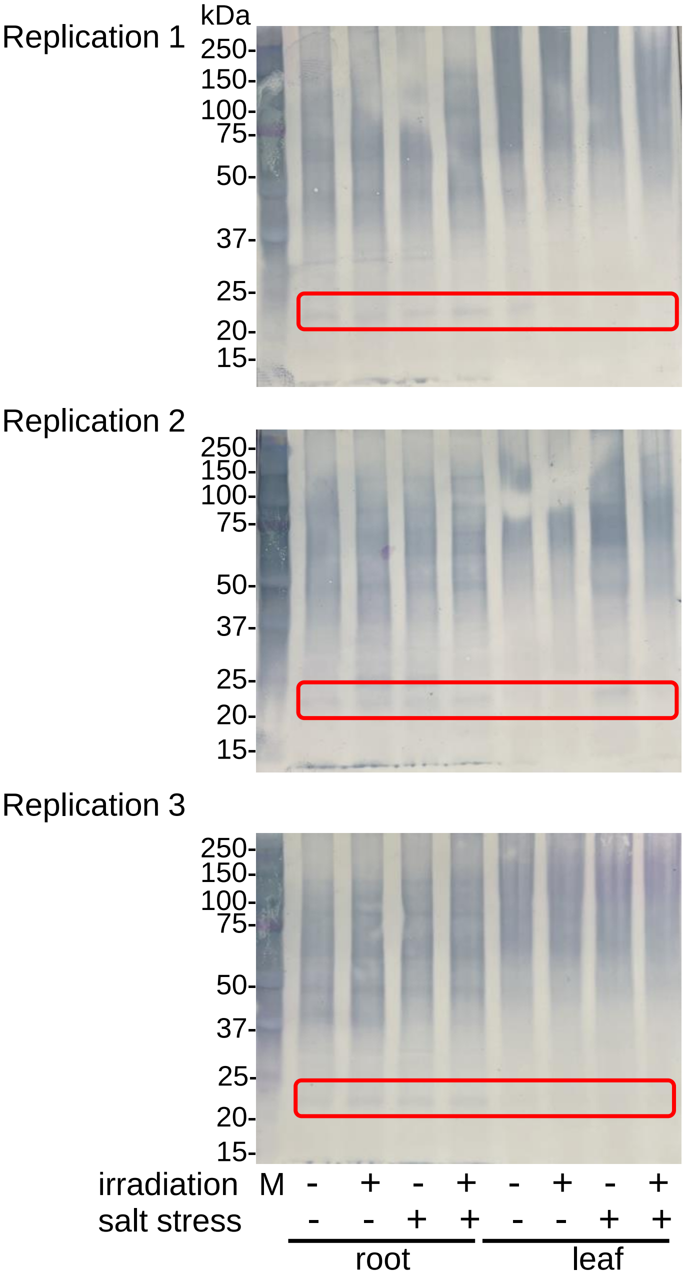

Figure S8. Blots of the entire membrane with anti-chitinase antibody, which is used in Figure 5.

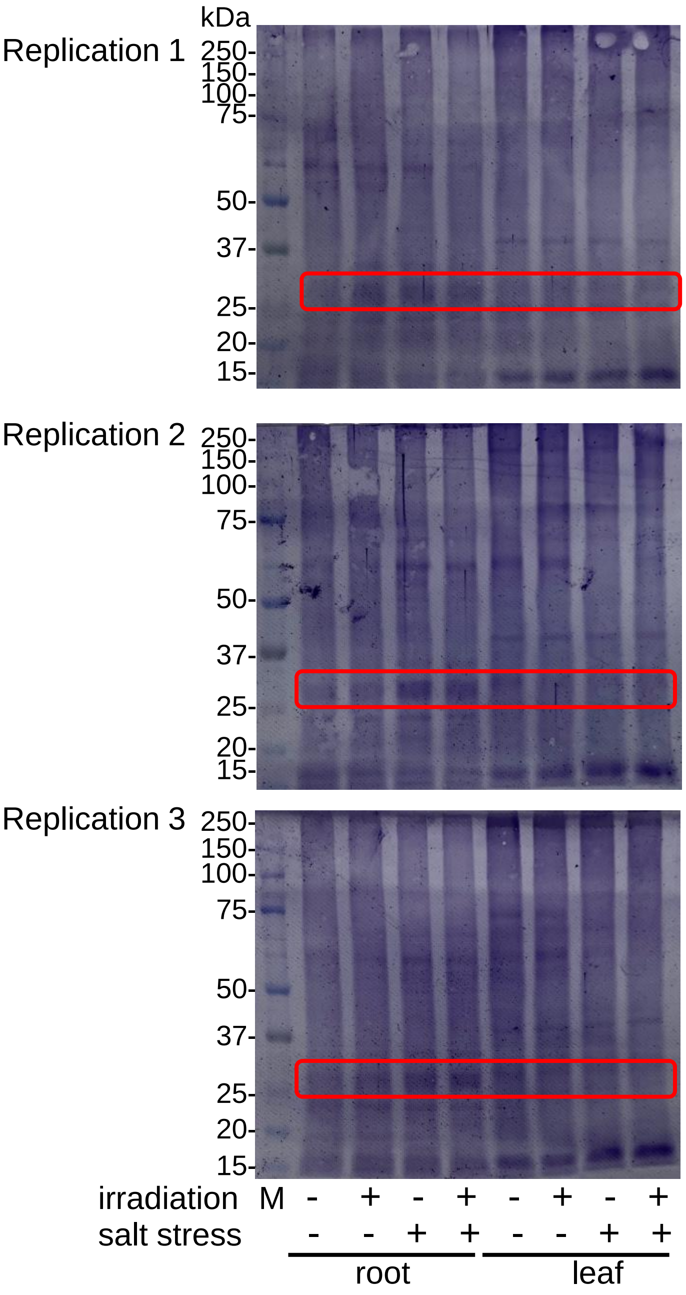

Figure S9: Blots of the entire membrane with anti-thaumatin antibody, which is used in Figure 5.

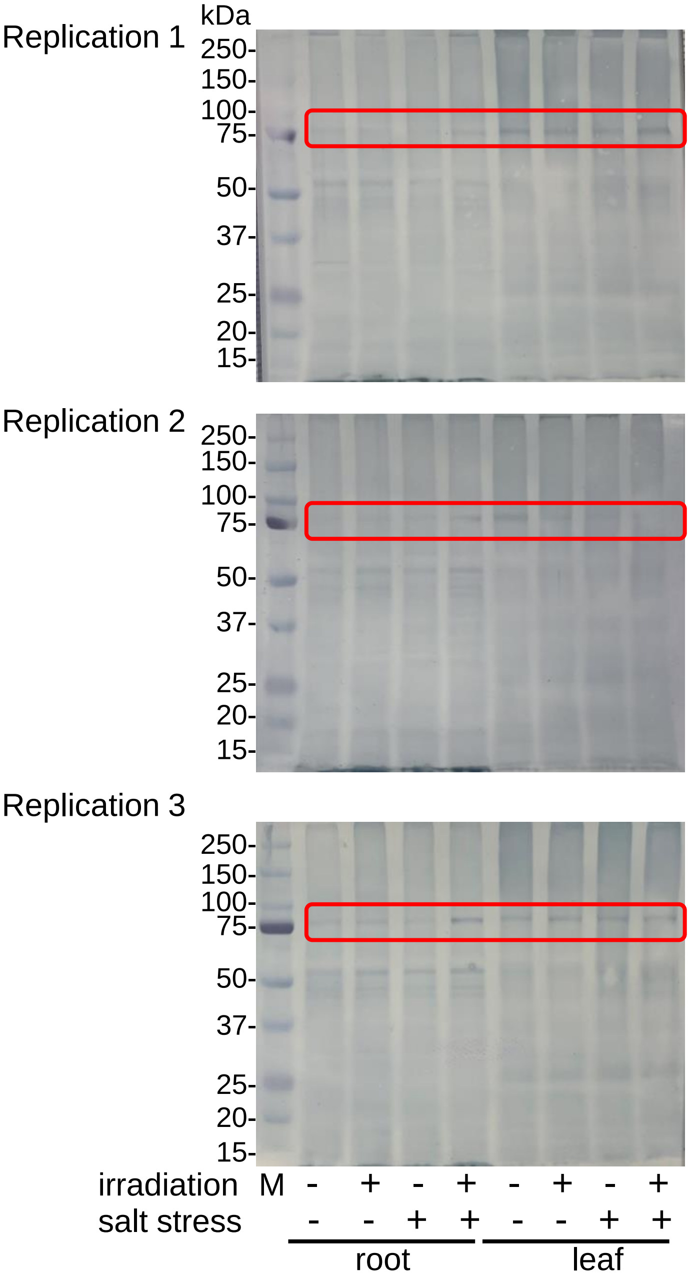

Figure S10: Blots of the entire membrane with anti-Bowman Birk proteinase inhibitor antibody, which is used in Figure 5.
