# Supplementary material for: Protein-Based Mechanism of Wheat Growth Under Salt Stress in Seeds Irradiated with Millimeter Waves
Source: Int J Mol Sci. 2024 Dec 30;26(1):253. doi: 10.3390/ijms26010253 (PMC11720253; doi:10.3390/ijms26010253)
Supplement: Supplementary file 1 [file ijms-26-00253-s001.zip › Supplemental Tables 1 and 2.pdf]

Table S1. Methods of MMW irradiation, protein identification using LC-MS/MS, and analysis of MS data.

	Method
MMW Irradiation	A Gunn oscillator (J. E. Caristrom, Chicago, IL, USA) was used as a MMW source, whose frequency range was 79 to 115 GHz and output power was 7 to 80 mW, depending on the output frequency. The Gunn oscillator was used in free running mode at 110 GHz. The electromagnetic waves emitted from the Gunn oscillator pass through an isolator after adjusting the output power by an attenuator; and then they were output to the free space via the horn antenna. The antenna pattern of the horn antenna had an aperture angle of 17 degrees on each side. By placing a 5 cm diameter petri dish containing the seeds of wheat (*Triticum aestivum* L. cultivar Nourin 61) at 15 cm from the horn antenna, the MMW radiation area fully covered the dish. To investigate the dependence of MMW irradiation on intensity, the irradiation time and the oscillation power were fixed at 20 mW for 20 min, respectively. The average intensity of the electromagnetic waves irradiated to the seeds was 0.25 mW/cm^2. The irradiated electromagnetic waves had a Gaussian distribution, with the maximum intensity at the center of the beam being 2.38 times of the average intensity and the minimum intensity at the rim of the beam being 0.32 times of the average intensity. The temperature rise of wheat was estimated to be well below 1 K.
Protein Identification using LC-MS/MS	The conditions of LC (EASY-nLC 1000; Thermo Fisher Scientific, San Jose, CA, USA) and MS (Orbitrap Fusion ETD MS; Thermo Fisher Scientific) were as follows. In brief, the peptides were loaded onto the LC system equipped with a trap column (Acclaim PepMap 100 C18 LC column, 3 μm, 75 μm ID x 20 mm; Thermo Fisher Scientific), equilibrated with 0.1% formic acid, and eluted with a linear acetonitrile gradient (0-35%) in 0.1% formic acid at a flow rate of 300 $nL\ min^{-1}$. The eluted peptides were loaded and separated on the column (EASY-Spray C18 LC column, 3 μm, 75 μm ID x 150 mm; Thermo Fisher Scientific) with a spray voltage of 2 kV (Ion Transfer Tube temperature: 275°C). The peptide ions were detected using MS in the data-dependent acquisition mode with the installed Xcalibur software (version 4.0; Thermo Fisher Scientific). Full-scan mass spectra were acquired in the MS over 375-1500 m/z with resolution of 120000. The most intense precursor ions were selected using the Top Speed acquisition algorithm for collision-induced fragmentation in the linear ion trap at normalized collision energy of 35%. Dynamic exclusion was employed within 60 sec to prevent repetitive selection of peptides.
Analysis of MS Data	The MS/MS searches were carried out using MASCOT (version 2.6.2; Matrix Science, London, UK) and SEQUEST HT search algorithms against the Arabidopsis Thaliana (UniProtKB TaxID=3702) (version 2021-02) and Triticum aestivum (SwissProt TaxID =4565) (version 2021-02) using Proteome Discoverer (version 2.4; Thermo Fisher Scientific). The workflow was as follows. The workflow for both algorithms included spectrum files RC, spectrum selector, MASCOT, SEQUEST HT search nodes, percolator, ptmRS, and minor feature detector nodes. Oxidation of methionine was set as a variable modification and carbamidomethylation of cysteine was set as a fixed modification. Mass tolerances in MS and

	MS/MS were set at 10 ppm and 0.6 Da, respectively. Trypsin was specified as protease and a maximum of 2 missed cleavage was allowed. Target-decoy database searches used for calculation of false discovery rate, which was set at 1% for peptide identification.
Differential Analysis of Proteins using MS Data	Label-free quantification using precursor-ions quantifiler nodes and principal-component analysis were performed with Proteome Discoverer 2.4. For differential analysis of the relative abundance of peptides and proteins between samples, the free software PERSEUS (version 1.6.15.0) was used. The workflow was as follows. Abundances of proteins and peptides abundances were transferred into log2 scale. Three biological replicates of each sample were grouped and a minimum of 3 valid values were required in at least one group. Normalization of the abundances was performed to subtract the median of each sample. Missing values were imputed based on a normal distribution (width = 0.3, down-shift = 1.8). Significance was assessed using Student's t-test analysis. The sequences of the differentially accumulated proteins were subjected to a BLAST query against the gene-ontology database (http://www.geneontology.org/).

Table S2. Morphological effect on MMW irradiated wheat treated with salt stress.

length

		mm	mean	± SE	± SD
root	Nontreatment	unirradiated	42.4	2.66	1.54
		MMW irradiation	39.1	1.75	1.01
	Salt stress	unirradiated	32.9	3.27	1.89
		MMW irradiation	38.9	0.21	0.12
leaf	Nontreatment	unirradiated	55.0	5.44	3.14
		MMW irradiation	50.8	4.36	2.51
	Salt stress	unirradiated	52.8	8.23	4.75
		MMW irradiation	47.9	1.96	1.13

fresh weight

		mg	mean	± SE	± SD
root	Nontreatment	unirradiated	39.6	2.70	1.56
		MMW irradiation	42.6	3.10	1.79
	Salt stress	unirradiated	34.2	3.25	1.88
		MMW irradiation	41.7	1.45	0.84
leaf	Nontreatment	unirradiated	46.6	2.85	1.65
		MMW irradiation	46.4	1.61	0.93
	Salt stress	unirradiated	43.6	3.24	1.87
		MMW irradiation	38.9	0.50	0.29

Wheat seeds were irradiated with or without MMW and sown. For nontreated groups, wheat seedlings were collected 5 days after sowing. For salt-stress groups, 3-day-old wheats were subjected to salt stress for 2 days and collected
